# Wine- and stir-frying processing of Cuscutae Semen enhance its ability to alleviate oxidative stress and apoptosis via the Keap 1-Nrf2/HO-1 and PI3K/AKT pathways in H_2_O_2_-challenged KGN human granulosa cell line

**DOI:** 10.1186/s12906-024-04491-5

**Published:** 2024-05-15

**Authors:** Yusha Liang, Yun Shi, Rong Guo, Changli Xu, Mian Fu, Jinyang Shen, Xun Gao, Weidong Li, Kunming Qin

**Affiliations:** 1 School of Pharmacy, Jiangsu Ocean University, Lianyungang, 222005 China; 2 Department of Pharmacy, Xinyi People’s Hospital, Xinyi Jiangsu, 221400 China; 3 Engineering Research Center of State Ministry of Education for Standardization of Chinese Medicine Processing, Nanjing University of Chinese Medicine, Nanjing, 210023 China

**Keywords:** Crude Cuscutae Semen (CCS), Wine-processed Cuscutae Semen (WCS), Stir-frying-processed Cuscutae Semen (SFCS), Human granulosa cell line KGN, Premature ovarian insufficiency (POI), Oxidative stress, Apoptosis

## Abstract

**Background:**

Cuscutae Semen (CS) has been prescribed in traditional Chinese medicine (TCM) for millennia as an aging inhibitor, an anti-inflammatory agent, a pain reliever, and an aphrodisiac. Its three main forms include crude Cuscutae Semen (CCS), wine-processed CS (WCS), and stir-frying-processed CS (SFCS). Premature ovarian insufficiency (POI) is a globally occurring medical condition. The present work sought a highly efficacious multi-target therapeutic approach against POI with minimal side effects. Finally, it analyzed the relative differences among CCS, WCS and SFCS in terms of their therapeutic efficacy and modes of action against H_2_O_2_-challenged KGN human granulosa cell line.

**Methods:**

In this study, ultrahigh-performance liquid chromatography (UPLC)-Q-ExactiveTM Orbitrap-mass spectrometry (MS), oxidative stress indices, reactive oxygen species (ROS), Mitochondrial membrane potential (MMP), real-time PCR, Western blotting, and molecular docking were used to investigate the protective effect of CCS, WCS and SFCS on KGN cells oxidative stress and apoptosis mechanisms.

**Results:**

The results confirmed that pretreatment with CCS, WCS and SFCS reduced H_2_O_2_-induced oxidative damage, accompanied by declining ROS levels and malondialdehyde (MDA) accumulation in the KGN cells. CCS, WCS and SFCS upregulated the expression of antioxidative levels (GSH, GSH/GSSG ratio, SOD, T-AOC),mitochondrial membrane potential (MMP) and the relative mRNA(Nrf2, Keap1, NQO-1, HO-1, SOD-1, CAT). They inhibited apoptosis by upregulating Bcl-2, downregulating Bax, cleaved caspase-9, and cleaved caspase-3, and lowering the Bax/Bcl-2 ratio. They also exerted antioxidant efficacy by partially activating the PI3K/Akt and Keap1-Nrf2/HO-1 signaling pathways.

**Conclusions:**

The results of the present work demonstrated the inhibitory efficacy of CCS, WCS and SFCS against H_2_O_2_-induced oxidative stress and apoptosis in KGN cells and showed that the associated mechanisms included Keap1-Nrf2/HO-1 activation, P-PI3K upregulation, and P-Akt-mediated PI3K-Akt pathway induction.

**Supplementary Information:**

The online version contains supplementary material available at 10.1186/s12906-024-04491-5.

## Background

In premature ovarian insufficiency (POI), the ovarian reserve is depleted before the age of 40 years. This disorder is characterized by sterility, amenorrhea or oligomenorrhea, hypoestrogenism, and elevated serum follicle-stimulating hormone (FSH) levels (> 25 mIU/mL). Women suffering from prolonged POI are at increased risk of cardiovascular diseases (CVDs), neurological impairment, psychological dysfunction, and osteoporosis [1]. The granulosa cell layer in the follicles mediates metabolism, material transport, and signal transduction in oocytes. Granulosa cells also play vital roles in follicular growth, proliferation, differentiation, atresia/ovulation, and corpus luteum formation [2]. Granulosa cell quality influences female fertility [3]. However, no efficacious therapies are currently available for POI, and the pathogenesis of this disorder remains poorly understood.

Cuscutae Semen (CS) has been prescribed in traditional Chinese medicine (TCM) for millennia. As wine- and stir-frying-processing alter the pharmaceutical effects of CS, these modified forms of the medicine have been administered for applications that clinically differ from those of CS. The earliest known record of Cuscutae Semen (CS; dry mature seeds of *Cuscuta australis* R. Br. or *Cuscuta chinensis* Lam.; family Convolvulaceae) dates back to the “Agriculture God’s Canon of Materia Medica” ca. 2,000 years ago. In this text, CS was designated a superior-grade drug. Presently, it is widely employed in Traditional Chinese Medicine (TCM) practices to nourish vital organs such as liver and kidney, treat impotence and seminal emission, prevent miscarriage, and enhance vision [4, 5]. Modern pharmacological experimentation demonstrated that CS reverses reductions in testosterone levels and androgen receptor gene expression, is immunomodulatory, hepatoprotective, antioxidant, anti-inflammatory, and anti-aging, and enhances memory by inducing PC12 cell differentiation [6–8]. Prior research showed that CCS supplementation invigorates internal organs such as the kidneys, and salt pretreatment potentiates it [9]. Our previous study indicated that heating transformed CCS into SFCS, and a hierarchical cluster analysis (HCA) clearly distinguished them both. A principal component analysis (PCA) disclosed that the levels of neochlorogenic acid, cryptophyllogenic acid, caffeic acid, quercetin, isorhamnetin, and kaempferol significantly differed between CCS and SFCS [10]. Though stir-frying adjusted the curative effect of CS, it was nonetheless difficult to distinguish among crude Cuscutae Semen (CCS), wine-processed Cuscutae Semen (WCS), and stir-frying-processed Cuscutae Semen (SFCS) in clinical practice. Moreover, little is known about the impact of CCS, WCS and SFCS on oxidative stress and apoptosis in POI. In 2023, the latest research shows that Dingkun Pill modulate ovarian function in premature ovarian insufficiency mice by regulating PI3K/AKT signaling pathway [11]. It also laid a foundation for the mechanism and target of treatment of premature ovarian failure before and after CS processing.

Reactive oxygen species (ROS) are the major chemical mediators of oxidative stress. Low ROS levels are essential for certain biological functions such as ovulation and luteolysis [12]. Nevertheless, excessive ROS production is detrimental to cell function and is associated with various human diseases such as diabetes [13], CVDs [14], and neuropathies [15]. POI is characterized by elevated serum ROS and oxidative stress marker levels [16, 17]. Severe oxidative stress may impair ovarian function [18]. However, the roles of oxidative stress in the etiology of POI have received limited research attention. The granulosa-like tumor cell line designated KGN is steroidogenic and is regarded as a reliable in vitro model of human granulosa cells [19]. H_2_O_2_-challenged KGN cells have been extensively used for in vitro experimentation involving POI [20].

Oxidative stress occurs when there is an elevated ratio of oxidants to antioxidants resulting in the disruption of redox signaling and the induction of molecular and cellular damage [21]. As excessive ROS cannot be eliminated fast enough, they can accumulate and interfere with the normal cellular redox state [22]. *N*-acetylcysteine (NAC) has mucolytic efficacy and potent antioxidant activity. It stimulates glutathione biosynthesis, promotes detoxification, and scavenges free radicals [23]. Oxidant and antioxidant systems are in dynamic equilibrium under normal physiological conditions. The antioxidant system consists of enzymes such as superoxide dismutase (SOD) and catalase (CAT) as well as non-enzymatic substances including glutathione (GSH), oxidized glutathione disulfide (GSSG), melatonin, ascorbic acid (vitamin C), vitamin E (mixed tocopherols), and copper, zinc, and selenium cations. The total antioxidant capacity (T-AOC) is another important indicator of redox balance. When ROS are overproduced or antioxidant biosynthesis decreases, the redox reactions go into dysequilibrium and the body enters oxidative stress (OS) [24]. OS causes pathological damage to the ovarian microenvironment including meiotic arrest in the oocytes, granulosa cell apoptosis, corpus luteum dysfunction, and accelerated ovary aging. However, antioxidant supplementation improves the OS and ovary function.

Antioxidant systems respond to the oxidative damage caused by ROS via several signaling pathways. Nuclear factor (erythroid-derived 2)-like 2 (Nrf2) is a major transcription factor (TF) associated with the oxidative stress response [25, 26]. Nrf2 is a member of the Cap’n’Collar (CNC) subfamily of basic leucine zipper (bZIP) TFs. It comprises seven conserved Nrf2-ECH homology (Neh) domains with different functions including the modulation of Nrf2 stability and contribution to the central role of Nrf2 in the redox system. Kelch-like-ECH-associated protein 1 (Keap1) regulates Nrf2 activity. In steady-state, Keap1 binds Nrf2 via its *C*-terminal domain and anchors it in the cytoplasm at a low basal protein level. The resultant Keap1-Cullin (CUL) 3-RING-box protein mediates Nrf2 ubiquitination and degradation via the 26S proteasome [27]. Oxidative stress inactivates Keap1, and newly synthesized Nrf2 translocates to the nucleus where it induces the transcription of antioxidant heme oxygenase 1 (HO-1) and quinine oxidoreductase 1 (NQO1) genes [28]. The mechanism by which oxidative stress activates Nrf2 is unknown. Nevertheless, a few earlier studies showed that upstream phosphatidylinositol-3-kinase (PI3K)/Akt activates Nrf2 and its downstream proteins such as HO-1 [29, 30].

The present study aimed to (1) establish whether CCS, WCS and SFCS inhibit oxidative stress and apoptosis in H_2_O_2_-challenged KGN cells and (2) elucidate their modes of action in POI treatment.

## Material and methods

### Materials and reagents

The materials used in this study included Cuscuta Semen (No. 20201201; Anhui Tonghuatang Chinese Herbal Decoction Pieces Co. Ltd., Anhui, China), *N*-acetylcysteine (NAC; No. ST1546, Beyotime Biotechnology, Shanghai, China), H_2_O_2_ (No. 20201201, Shanghai Selleck Chemicals Co. Ltd., Shanghai, China), fetal bovine serum (FBS; (No. 164210–500, Procell Life Science & Technology Co. Ltd., Wuhan, China), Dulbecco’s modified Eagles’s medium/F12 medium (DMEM/F12; No. VCM12500, VICMED, Xuzhou, China), Cell Counting Kit-8 (CCK-8; No. C6005; NCM Biotech, Suzhou, China), ROS Detection Kit (No. S0033S; Beyotime Biotechnology), Malondialdehyde (MDA) Assay Kit (No. S0131S; Beyotime Biotechnology), Enhanced Bicinchonic Acid (BCA) Protein Assay Kit (No. P0010; Beyotime Biotechnology), Superoxide Dismusate (SOD) Assay Kit (No. S0109; Beyotime Biotechnology), Glutathione (GSH) and Oxidized Glutathione (GSSG) Assay Kit (No. S0053; Beyotime Biotechnology), Mitochondrial Membrane Potential with Mito® Tracker Red CMXRos (No. C1071S; Beyotime Biotechnology), Total Antioxidant Capacity Assay Kit (No. AK351; Bioss Inc., Beijing, China), TRIzol reagent (No. R401-01; Vazyme Biotech, Shanghai, China), RT Master Mix for qPCR II (No. HY-K0511A; MedChemExpress (MCE), Monmouth Junction, NJ, USA), SYBR Green Real MasterMix (No. HY-K0523; MCE)The primary antibodies anti-Keap 1 (No. TA5266F), anti-NQO1 (No. T56710F), anti-Bcl-2 (No. T40056F), anti-Nrf2 (No. T55136F), anti-HO 1 (No. T55113F), anti-Bax (No. T40051F), anti-PI3K/p85-α (Nos. T40115F and T40116F), anti-phospho-Akt (Ser473) (No. T40067F), anti-Akt1/2/3 (No. T55561F), and anti-PI3K/p85-α (T40115F) were purchased from Abmart (Berkeley Heights, NJ, USA). The primary antibodies anti-SOD2 (No. WL02506), anti-cleaved caspase-9 (No. WL01838), and anti-cleaved caspase 3 (No. WL02117) were purchased from Wanlei Biotechnology Co. Ltd. (Shanghai, China). The primary antibody anti-β-actin (No. 66009–1-Ig) was purchased from Proteintech Group (Rosemont, IL, USA). The secondary antibodies anti-rabbit immunoglobulin G (IgG) (H + L) (No. SA00001-2) and anti-mouse IgG (H + L) (No. SA00001-1) were purchased from Proteintech Group.

### CCS, WCS and SFCS extract processing

One-half gram (0.5 g) of each of the CCS, WCS and SFCS powders was precisely weighed out, extracted by ultrasonication with 20 mL of 80% (v/v) ethanol for 30 min, and centrifuged at 13,000 rpm for 5 min. Each solution was then transferred to a 25-mL volumetric flask and the volume was made up to the mark with 80% (v/v) methanol. Each solution was then concentrated and freeze-dried to powder under vacuum and each powder was stored at -80 °C until the subsequent experiments.

### Ultrahigh-performance Q-exactive™ mass spectrometry (UPLC-Q-Exactive.™ Orbitrap-MS)

UPLC-Q-Exactive™ Orbitrap-MS was conducted in a Thermo Fisher U3000 UPLC fitted with an online degasser, a quaternary gradient pump, a column temperature chamber, an automatic sampler, and a Q Exactive™ Plus Orbitrap ionization source (Thermo Fisher Scientific, Waltham, MA, USA). Chromatographic separation was performed with a Waters ACQUITY UPLC HSS T3 C18 column (2.1 mm × 100 mm, 1.8 μm; Waters Corporation, Milford, MA, USA). UV detection of the UHPLC fractions was executed with a U3000 3D field DAD detector. The analytical column temperature was fixed at 35 °C, and the injection volume was 10 μL. Gradient elution was performed using 0.1% (v/v) formic acid in water (solvent A) and acetonitrile (solvent B). The flow rate was set to 0.3 mL/min, and the gradient elution program was as follows: 0–10 min, 100% A; 10–25 min, 70–60% A; 25–30 min, 60–50% A; 30–40 min, 50–30% A; 40–45 min, 30–0%; 45–60 min, 0% A; 60–60.5 min, 0–100% A; 60.5–70 min, 100% A. For the MS, the positive and negative ion modes were in the range of m/z 100–1,200, the sheath and auxiliary gas flow rates were 40 arb and 15 arb, respectively, the capillary and Aux gas heater temperatures were 320 °C and 350 °C, respectively, the positive spray voltage was 3.2 kV, and the MS and MS/MS resolutions were 70,000 and 17,500, respectively. A Cuscutae Semen composition database was established based on a literature review and a TCM natural products database to summarize and analyze the unknown substances in CCS, WCS and SFCS.

### Cell culture and treatment

The human ovarian granule KGN cell line was purchased from Punosai Company (Wuhan, China) and cultured in DMEM/F12 medium containing 100 U/mL penicillin, streptomycin, and 10% (v/v) FBS in an incubator at 37 °C, 75% RH, and 5% CO_2_. The groups to which the KGN cells were assigned included the normal control (CON); model (MOD, 400 μΜ H_2_O_2_); positive control (NAC, 5 mM NAC + 400 μΜ H_2_O_2_); crude CS (CCS, 0.1 mg/mL CS extract + 400 μΜ H_2_O_2_ and 0.3 mg/mL CS extract + 400 μΜ H_2_O_2_), CCS low-dose (CCS-L, 0.1 mg/mL), CCS high-dose (CCS-H, 0.3 mg/mL); wine-processed CS (WCS, 0.1 mg/mL WCS extract + 400 μΜ H_2_O_2_ and 0.3 mg/mL WCS extract + 400 μΜ H_2_O_2_), WCS low-dose (WCS-L, 0.1 mg/mL), WCS high-dose (WCS-H, 0.3 mg/mL); stir-frying-processed CS (SFCS, 0.1 mg/mL SFCS extract + 400 μΜ H_2_O_2_ and 0.3 mg/mL SFCS extract + 400 μΜ H_2_O_2_), SFCS low-dose (SFCS-L, 0.1 mg/mL), and SFCS high-dose (SFCS-H, 0.3 mg/mL).

### Cell viability assay

KGN cells were seeded in 96-well flat-bottomed plates at 4 × 10^4^/well and exposed to various CCS, WCS and SFCS concentrations for 24 h to establish the safe CCS, WCS and SFCS concentration range. The cells were then treated with different H_2_O_2_ concentrations for 2 h to determine the safe H_2_O_2_ exposure range. KGN cells were then seeded in 96-well plates at 4 × 10^4^/well and subjected to CCS, WCS, SFCS, and NAC for 24 h and then to H_2_O_2_ for 2 h. Cell viability was then measured with a CCK-8 Kit and an inverted microscope.

### MDA, SOD, T-AOC, GSH, GR, and GSSG measurements

Peroxidation of the KGN cell membrane lipids was assessed by measuring MDA production. The antioxidant ability was evaluated by detecting SOD, T-AOC, GSH, GSSG, and GR using commercially available kits and according to the manufacturers’ instructions.

### Intracellular ROS measurement

Intracellular ROS was measured with the ROS Assay Kit according to the manufacturer’s protocol. KGN cells exposed to CCS, WCS, SFCS, NAC, H_2_O_2_ and active oxygen-positive (AOP) were incubated with 10 μM of the 2`,7`-dichlorofluorescein diacetate (DCFH-DA) fluorescent probe at 37 °C for 30 min, washed twice with pre-cooled phosphate-buffered saline (PBS), and photographed under a fluorescence microscope.

### Mitochondrial membrane potential detection

KGN cell apoptosis was detected with Mito® Tracker Red CMXRos (No. C1071S; Beyotime Biotechnology). The treated KGN cells were collected in 0.25% (w/v) trypsin (No. VC2004; VICMED), washed twice with PBS, resuspended in 188 μL PBS, incubated with 2 μL Mito® Tracker Red CMXRos and 5 μL Hoechst33342 in the dark at 37 °C for 30 min, quantified with ImageJ software (National Institutes of Health [NIH], Bethesda, MD, USA), and photographed under a fluorescence microscope.

### Gene expression analysis

TRIzol reagent (Vazyme Biotech) was used to extract the RNA from the KGN cells. RNA purity and concentration were measured in a NanoDrop 2000 spectrophotometer (Thermo Fisher Scientific). DNA was removed with RNase-Free DNase (MCE) and the RNA was reverse-transcribed to cDNA with MonScript RTIII Super Mix (MCE). The output was then statistically analyzed in a Step One Plus Detection System (Thermo Fisher Scientific). The data acquired were based on β-actin as the internal reference, and the relative gene expression levels were computed by the 2^−ΔΔCt^ method. The specific primers used are listed in Table 1.

**Table 1:** Primer sequences used in Real-Time PCR analyses

Gene	Forward Sequence	Reverse Sequence	NCBI No
Nrf2	TCACACGAGATGAGCTTAGGGCAA	TACAGTTCTGGGCGGCGACTTTAT	NM_010902.4
Keap1	CAGCAACTCTGTGACGTGACC	TCAATAAGCCTTTCCATGACCT	NM_016679.4
SOD1	TGGTTTGCGTCGTAGTCTCC	CTTCGTCGCCATAACTCGCT	NM_000454.4
HO-1	ATGGCCTCCCTGTACCACATC	TGTTGCGCTCAATCTCCTCCT	NM_002133.2
CAT	CCATTATAAGACTGACCAGGGC	AGTCCAGGAGGGGTACTTTCC	NM_001752.3
Bcl-2	ATGTGTGTGGAGCGTCAACC	CAGAGACAGCCAGGAGAAATC	NM_000633.3
Bax	GAGCTGCAGAGGATGATTGCT	TGATCAGCTCGGGCACTTTA	NM_007527.3
β-actin	CAAGAGAGGTATCCTGACCT	TGATCTGGGTCATCTTTTCAC	NM_007393.5

### Western blotting

KGN cells were lysed with radioimmunoprecipitation (RIPA) buffer supplemented with phenylmethanesulfonyl fluoride (PMSF) and centrifuged at 12,000 rpm and 4 °C for 10 min. The protein concentration in the samples was detected by the BCA method (Beyotime Biotechnology). The proteins were separated by sodium dodecyl sulfate–polyacrylamide gel electrophoresis (SDS-PAGE) and transferred to a polyvinylidine difluoride (PVDF) membrane (EMD Millipore, Billerica, MA, USA). Each membrane was blocked, incubated at 4 °C overnight with the aforementioned primary antibodies, and probed with horseradish peroxidase (HRP)-conjugated secondary antibody at room temperature for 1 h. An enhanced chemiluminescence (ECL) system (VICMED) and luminescent solution were used to detect fluorescent bands in an imager, and the bands were quantitatively analyzed with ImageJ software.

### Statistical analysis

All data were analyzed with GraphPad Prism v. 9.5 (GraphPad Software, La Jolla, CA, USA) and presented as means ± standard deviation (SD). Multiple comparisons were made via one-way analysis of variance (ANOVA) followed by Tukey’s post-hoc test. *P* < 0.01 or *P* < 0.05 indicated a statistically significant difference.

## Results

### Chemical constituents of CCS, WCS and SFCS

**Fig. 1:**
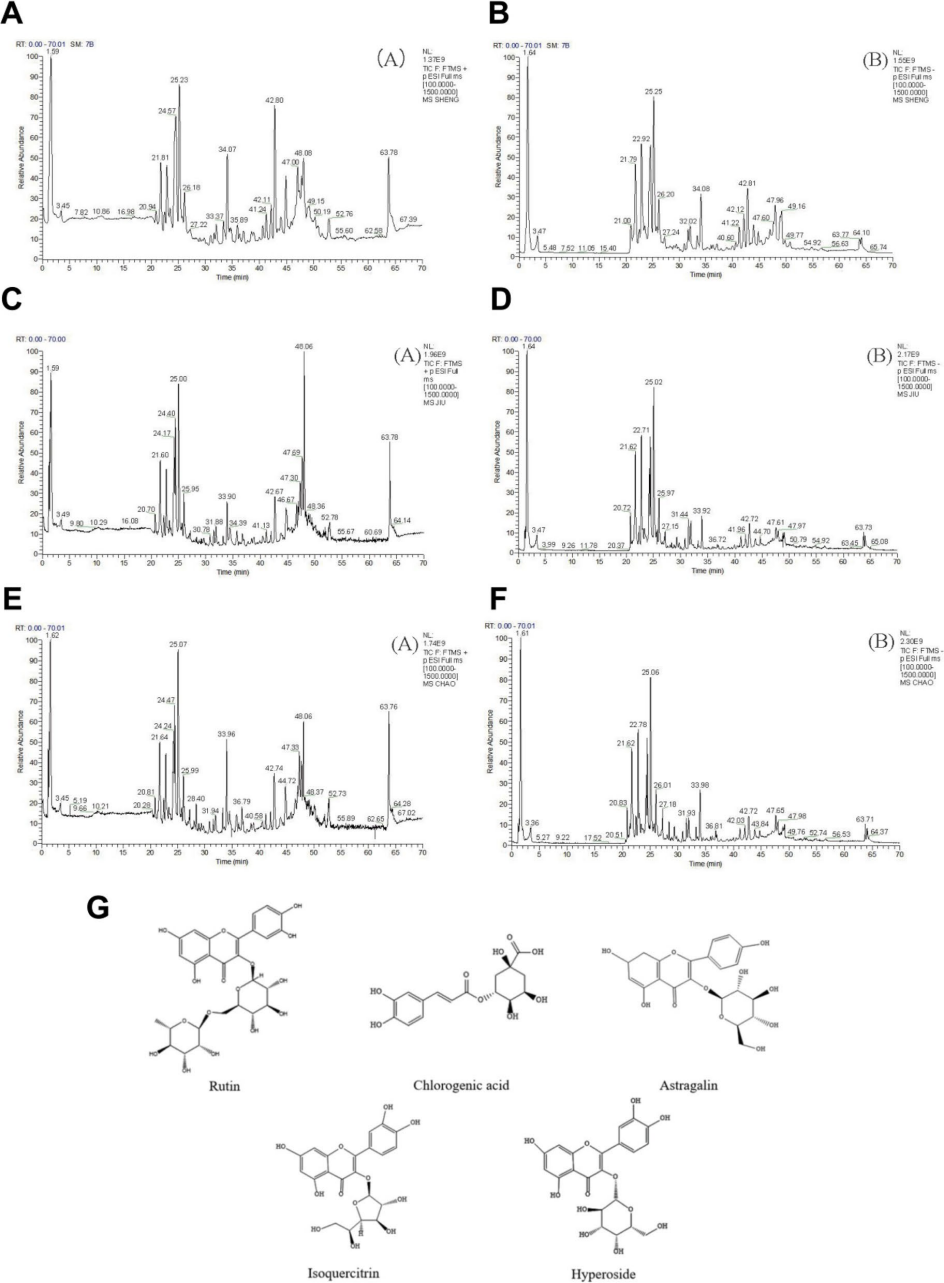
Chemical constituents in CCS, WCS, and SFCS. **A**, **B** Total ion chromatogram of CCS extracts in positive (**A**) and negative (**B**) ion mode. **C**, **D** Total ion chromatogram of WCS extracts in positive (**A**) and negative (**B**) ion mode. **E**, **F** Total ion chromatogram of SFCS extracts in positive (**A**) and negative (**B**) ion mode. **G** Chemical structures of CCS, WCS, and SFCS

UPLC-Q-Exactive™ Orbitrap-MS was used to analyze the CCS, WCS and SFCS extracts. Total ion chromatograms in negative and positive ion modes for CCS, WCS and SFCS are shown in Fig. 1A–F. The five major bioactive compounds were identified as rutin, chlorogenic acid, astragalin, isoquercitrin, and hyperoside (Fig. 1G). The top 30 ingredients were detected in CCS, WCS and SFCS, and they included mainly flavonoids, phenolic acids, and lignans. They are listed in Table 2 and the last 51 ingredients were listed in Supplementary Material 1.

**Table 2:** Principal component identifications of different processed products of Cuscuta Semen

No	Identification	Formula	theoretical value(*m/z*)	Error (ppm)	*t*R/min	Source	Quasi-molecular ion
1	Kaempferol	C_15_H_10_O_6_	286.0477	-0.15	31.51	CCS,WCS,SFCS	[M-H]^−^
2	Astragalin	C_21_H_20_O_11_	448.1006	-0.02	26.01	CCS,WCS,SFCS	[M-H]^−^
3	Isorhamnetin	C_16_H_12_O_7_	316.0583	0.05	31.85	CCS,WCS,SFCS	[M-H]^−^
4	Hyperoside	C_21_H_20_O_12_	464.0955	0.13	25.09	CCS,WCS,SFCS	[M-H]^−^
5	Quercetin	C_15_H_10_O_7_	302.0426	-0.2	29.34	CCS,WCS,SFCS	[M-H]^−^
6	Isochlorogenic acid C	C_25_H_24_O_12_	516.1268	0.12	26.40	CCS,WCS,SFCS	[M-H]^−^
7	Chlorogenic acid	C_16_H_18_O_9_	354.0950	-0.35	21.82	CCS,WCS,SFCS	[M-H]^−^
8	Cryptochlorogenic acid	C_16_H_18_O_9_	354.0950	-0.25	22.77	CCS,WCS,SFCS	[M + H]^+^
9	Quinic acid	C_7_H_12_O_6_	192.0633	-0.24	22.78	CCS,WCS,SFCS	[M + H]^+^
10	Isochlorogenic acid B	C_25_H_24_O_12_	516.1269	0.14	25.52	WCS,SFCS	[M + H]^+^
11	Caffeic acid	C_9_H_8_O_4_	180.0423	0.03	22.80	CCS,WCS,SFCS	[M + H]^+^
12	Neochlorogenic acid	C_16_H_18_O_9_	354.0951	-0.09	25.53	CCS,WCS,SFCS	[M + H]^+^
13	Apigenin	C_15_H_10_O_5_	270.0529	0.13	31.16	CCS,WCS,SFCS	[M-H]^−^
14	Pinoresinol	C_26_H_32_O_11_	520.1947	0.42	25.60	CCS,WCS,SFCS	[M + H]^+^
15	Ferulic acid	C_10_H_10_O_4_	194.0579	0.16	25.50	CCS,WCS,SFCS	[M + H]^+^
16	Manninotriose	C_18_H_32_O_16_	504.1691	0.17	1.68	CCS,WCS,SFCS	[M + H]^+^
17	p-Coumaric acid	C_9_H_8_O_3_	164.0475	0.66	24.90	CCS,WCS,SFCS	[M + H]^+^
18	Gallic acid	C_7_H_6_O_5_	170.0216	0.15	27.66	CCS,WCS,SFCS	[M + H]^+^
19	Abscisic acid	C_15_H_20_O_4_	264.1362	0.01	28.61	CCS,WCS,SFCS	[M-H]^−^
20	Arglabin	C_15_H_18_O_3_	246.1256	0.07	25.58	CCS,WCS,SFCS	[M + H]^+^
21	( +)-Pinoresinol	C_20_H_22_O_6_	358.1416	-0.21	25.60	CCS,WCS,SFCS	[M-H]^+^
22	Abietic acid	C_20_H_30_O_2_	302.2245	-0.27	47.45	WCS,SFCS	[M + H]^+^
23	Gentiopicrin	C_16_H_20_O_9_	356.1108	0.09	22.47	CCS,WCS,SFCS	[M + H]^+^
24	2-Hydroxy-4-methoxybenzaldehyde	C_8_H_8_O_3_	152.0474	0.50	24.78	CCS,WCS,SFCS	[M + H]^+^
25	Daidzin	C_21_H_20_O_9_	416.1106	-0.39	24.09	CCS,WCS,SFCS	[M + H]^+^
26	4-Hydroxybenzoic acid	C_7_H_6_O_3_	138.0317	0.11	22.30	CCS,WCS,SFCS	[M + H]^+^
27	Naringenin	C_15_H_12_O_5_	272.0684	-0.13	30.93	CCS,WCS,SFCS	[M + H]^+^
28	Daidzein	C_15_H_10_O_4_	254.0579	-0.11	28.49	CCS,WCS,SFCS	[M + H]^+^
29	Arbutin	C_12_H_16_O_7_	272.0898	0.68	6.26	CCS,SFCS	[M + H]^+^
30	Glycitein	C_16_H_12_O_5_	284.0684	-0.42	28.89	CCS,WCS,SFCS	[M + H]^+^

### Effects of CCS, WCS and SFCS on KGN cell viability

**Fig. 2:**
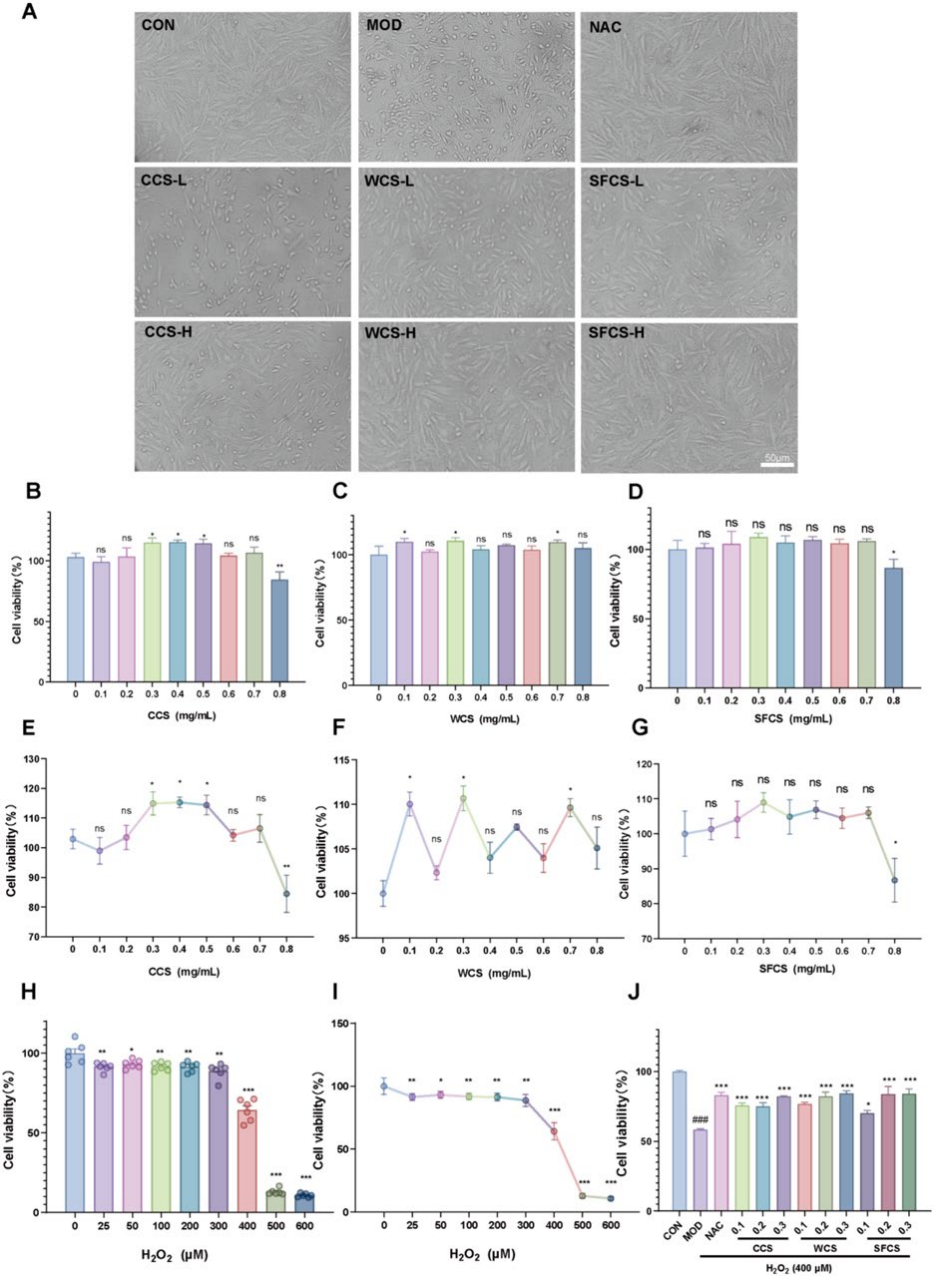
CCS, WCS, and SFCS mitigated injury to H_2_O_2_-challenged KGN cells. **A** Photographs of untreated KGN cells and those treated with CCS, WCS, SFCS, NAC, and H_2_O_2_. **B**–**J** Effect of CCS, WCS, SFCS, NAC, and H_2_O_2_ on KGN cell viability. **D** Effect of CCS on KGN cell viability. Data are means ± standard deviation (S.D.) (*n* ≥ 3). ^###^*P* < 0.001 vs. CON group; ^*^P < 0.05, ^**^P < 0.01, ^***^P < 0.001 vs. MOD group; ns, not significantly different from the MOD group

The effects of CCS, WCS, SFCS, NAC, and H_2_O_2_ on KGN cell viability were evaluated by CCK-8 assay. At 0.1–0.7 mg/mL, CCS, WCS and SFCS were not cytotoxic to KGN cells compared to the control. At 0.8 mg/mL, however, CCS was not cytotoxic but WCS and SFCS were significantly cytotoxic to KGN cells (Fig. 2C–E). KGN cells were subjected to various H_2_O_2_ concentrations (0–600 μM), and the one inducing oxidative damage was determined. Figure 2B shows that 400 μM H_2_O_2_ decreased KGN cell viability to ~ 40%. Hence, this H_2_O_2_ concentration was selected for the subsequent experiments. Figure 2F shows that pretreatment with CCS, WCS, SFCS, and NAC for 24 h alleviated damage to H_2_O_2_-challenged KGN cells. KGN cells were co-treated with 400 μM H_2_O_2_ and either 0.1 mg/mL or 0.3 mg/mL CCS, WCS and SFCS as well as 5 mM NAC. While both concentrations of CCS, WCS and SFCS alleviated injury to H_2_O_2_-challenged KGN cells at 24 h. Microscopic examination confirmed that CCS, WCS, SFCS, and NAC inhibited morphological damage to H_2_O_2_-challenged KGN cells (Fig. 2A). Moreover, WCS and SFCS exerted a stronger protective effect than CCS against H_2_O_2_-induced damage in KGN cells.

### CCS, WCS and SFCS decreased MDA and GSSG but increased T-AOC, SOD, GSH, GR, and GSH/GSSG ratio in H_2_O_2_-challenged KGN cells

**Fig. 3:**
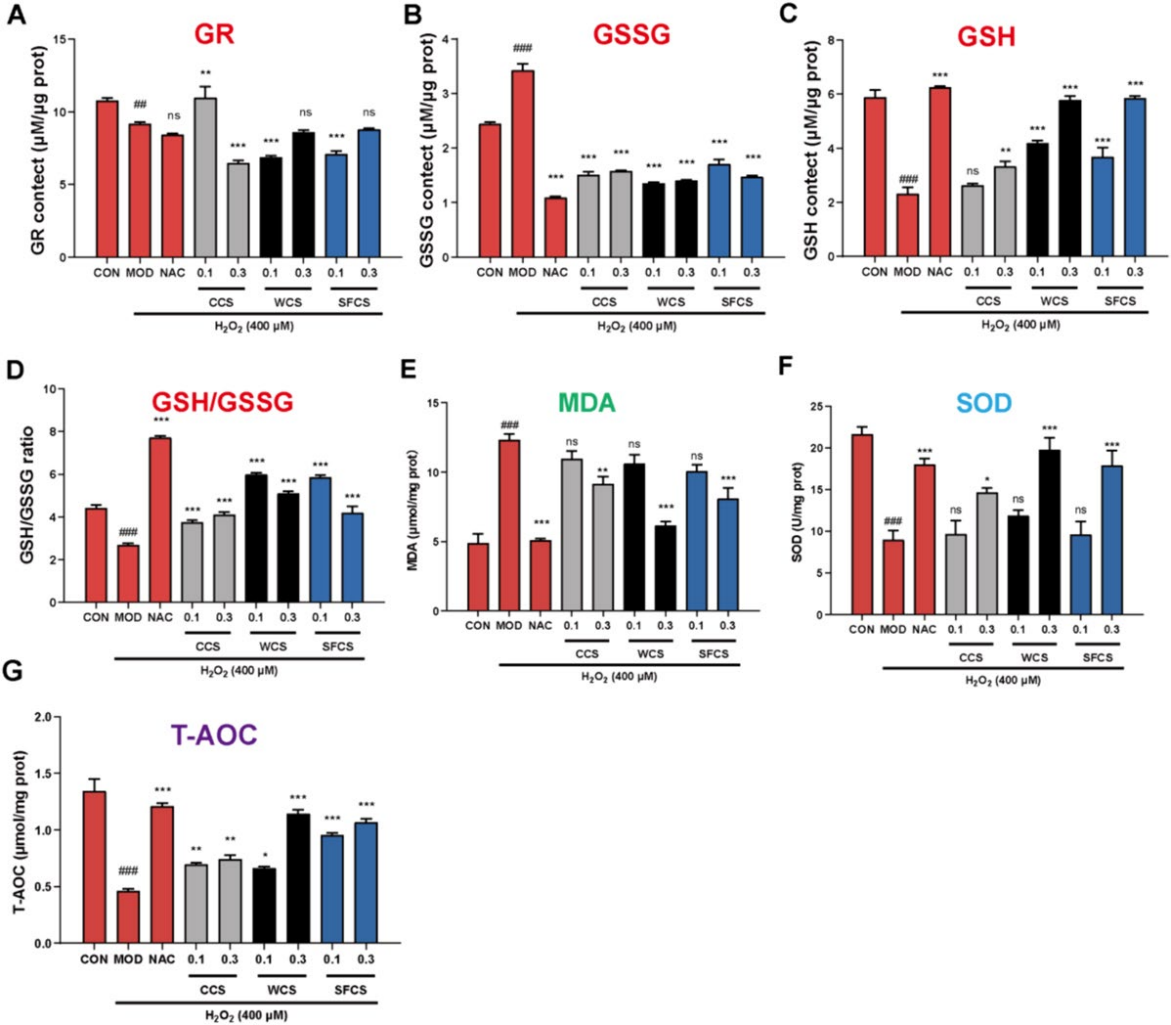
CCS, WCS, and SFCS offset redox imbalance in H_2_O_2_-challenged KGN cells. **A** GR levels in KGN cells. **B** GSSG levels in KGN cells. **C** GSH levels in KGN cells. **D** GSH/GSSG ratios in KGN cells. **E** MDA levels in KGN cells. **F** SOD levels in KGN cells. **G** T-AOC levels in KGN cells. Data are means ± S.D. (*n* ≥ 3). ^###^*P* < 0.001, ^##^*P* < 0.01 vs. CON group; ^*^P < 0.05, ^**^P < 0.01, ^***^P < 0.001 vs. MOD group; ns, not significantly different from the MOD group

The observed changes in the MDA, T-AOC, SOD, GSH, GR, and GSSG levels and the GSH/GSSG ratio in the KGN cells indicated that H_2_O_2_ exposure induced oxidative stress in them. In the MOD group, the MDA and GSSG levels had increased while SOD, T-AOC, GSH, and GR activity and the GSH/GSSG ratio had decreased compared to the CON group. However, the foregoing abnormal changes were partially rescued in response to the NAC, CCS, WCS and SFCS treatments. NAC and high concentrations of WCS and SFCS effectively attenuated the adverse effects of oxidative stress in the MOD group (Fig. 3A–G). The preceding results suggest that NAC inhibited oxidative stress in H_2_O_2_-challenged KGN cells while WCS and SFCS more effectively mitigated the inhibitory effect of oxidative stress on KGN cells than CCS. Hence, wine- and stir-frying processing enhance the antioxidant capacity of CS.

### CCS, WCS and SFCS increased mitochondrial activity in H_2_O_2_-challenged KGN cells

**Fig. 4:**
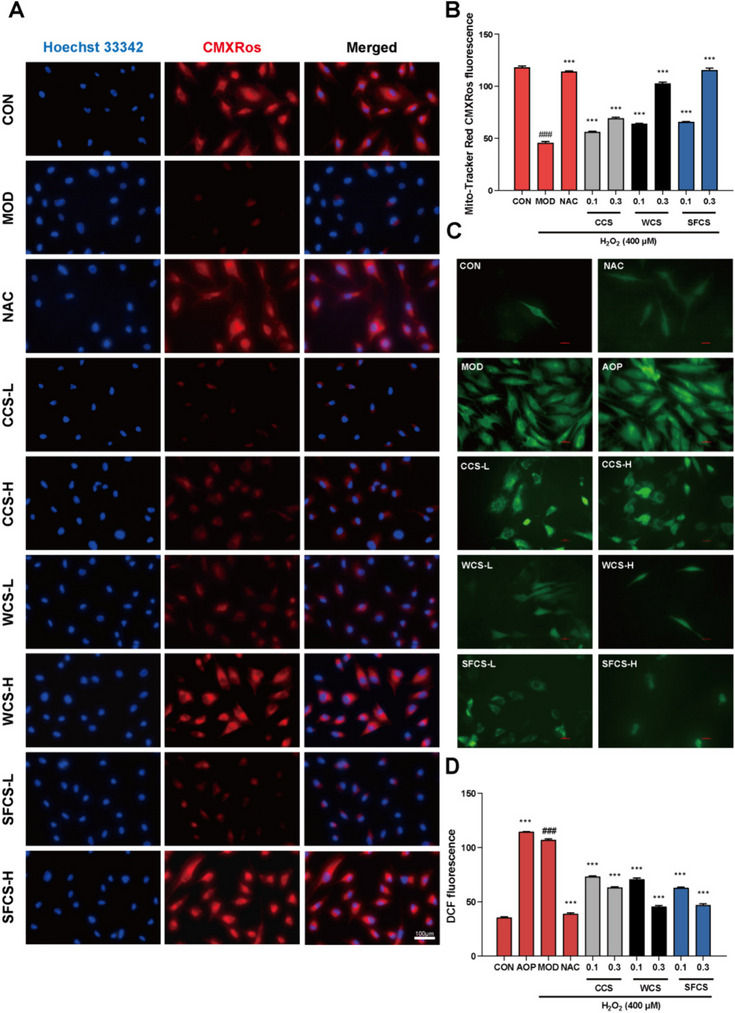
CCS, WCS, and SFCS increased mitochondrial activity in H_2_O_2_-challenged KGN cells. **A** Mitochondrial membrane potentials (MMP) in KGN cells were measured by CMXRos staining and fluorescence microscopy. **B** Quantification of CMXRos fluorescence. **C** ROS levels in KGN cells were measured by staining with DCFH-DA and fluorescence microscopy. **D** Quantification of DCF fluorescence. Data are means ± S.D. (*n* ≥ 3). ^###^*P* < 0.001 vs. CON group; ^***^P < 0.001 vs. MOD group

Abnormal ROS accumulation was observed in KGN cells exposed to H_2_O_2_. The fluorescence intensity was significantly higher in the H_2_O_2_-challenged KGN cells than in the untreated control. Pretreatment with NAC, WCS-H, and SFCS-H markedly decreased intracellular ROS whereas AOP, CCS-L, CCS-H, WCS-L, and SFCS-L had minimal impact on it (Fig. 4C and D).

The loss of mitochondrial membrane potential (MMP) in response to H_2_O_2_-induced cytotoxicity was measured by a Mito® Tracker Red CMXRos assay. A decrease in MMP indicates apoptosis. Figure 4A–B show that Mito® Tracker Red CMXRos aggregated and emitted red fluorescence in the CON group. The ratio of red/blue fluorescent positive cells was greatly decreased after H_2_O_2_ exposure, and pretreatments with CCS, WCS and SFCS significantly increased the ratio in a dose-dependent manner.

### Regulation of apoptosis-related protein and gene levels in H_2_O_2_-challenged KGN cells by CCS, WCS and SFCS

**Fig. 5:**
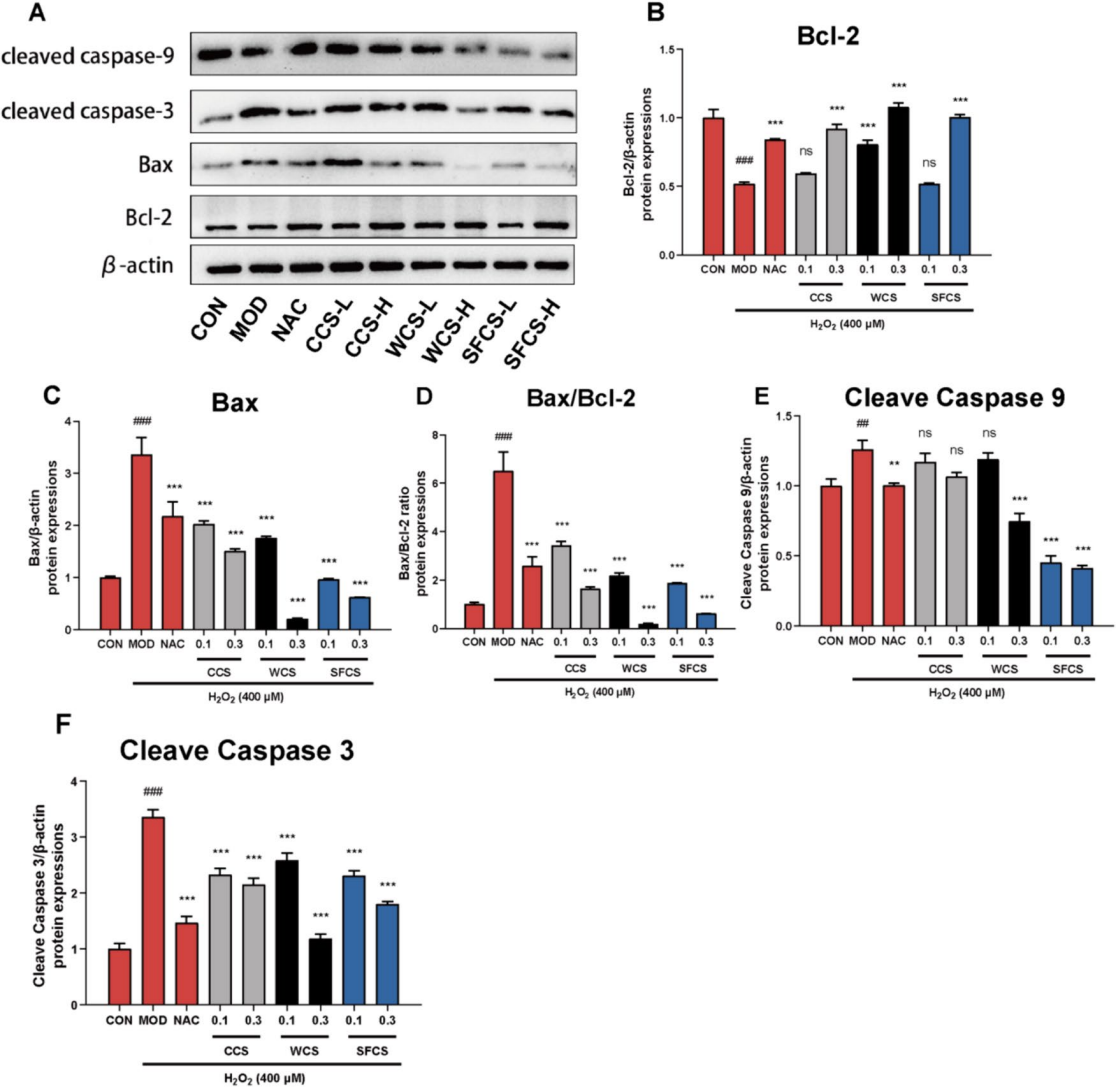
Regulation of apoptosis-related protein levels by CCS, WCS, and SFCS in H_2_O_2_-challenged KGN cells. **A**–**F** BcL-2, Bax, cleaved caspase 9, and cleaved caspase 3 protein levels and Bax/Bcl-2 ratios in KGN cells treated with CCS, WCS, and SFCS in the presence or absence of H_2_O_2_ treatment. Protein expression was normalized against β-actin. Data are means ± S.D. (*n* ≥ 3). ^###^*P* < 0.001, ^##^*P* < 0.01 vs. CON group; ^**^P < 0.01, ^***^P < 0.001 vs. MOD group; ns, not significantly different from the MOD group

**Fig. 6:**
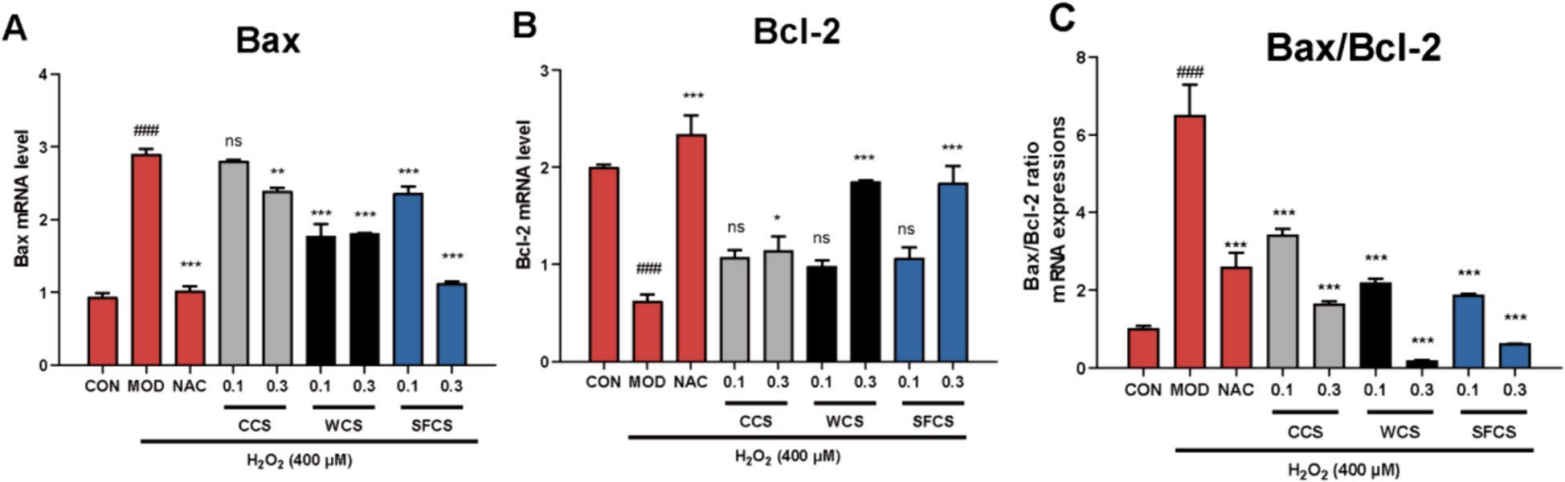
Regulation of apoptosis-related gene levels by CCS, WCS, and SFCS in H_2_O_2_-challenged KGN cells. **A**–**C** BcL-2 and Bax gene expression and Bax/Bcl-2 ratios in response to CCS, WCS, and SFCS in the presence or absence of H_2_O_2_ treatment. Gene expression was normalized against β-actin. Data are means ± S.D. (*n* ≥ 3). ^###^*P* < 0.001 vs. CON group; ^*^P < 0.05, ^**^P < 0.01, ^***^P < 0.001 vs. MOD group; ns, not significantly different from the MOD group

Various stressors cause mitochondrial outer membrane permeabilization (MOMP), thereby initiating mitochondrial apoptosis. The expression levels of the aforementioned apoptosis-related proteins and their corresponding genes were assessed to determine the effects of CCS, WCS and SFCS on KGN cells. The levels of pro-apoptotic Bax, cleaved caspase-9, and cleaved caspase-3 were lower while the level of anti-apoptotic Bcl-2 was higher in the KGN cells treated with CCS, WCS and SFCS than in those challenged with H_2_O_2_, and the magnitudes of these differences were dose-dependent (Fig. 5A–F). The qPCR confirmed that CCS, WCS and SFCS downregulated Bax mRNA and upregulated Bcl-2 mRNA (Fig. 6A–C). WCS and SFCS were more effectively anti-apoptotic at 0.3 mg/mL than at 0.1 mg/mL.

### CCS, WCS and SFCS activate the Keap1-Nrf2/HO-1 pathway in H_2_O_2_-challenged KGN cells

**Fig. 7:**
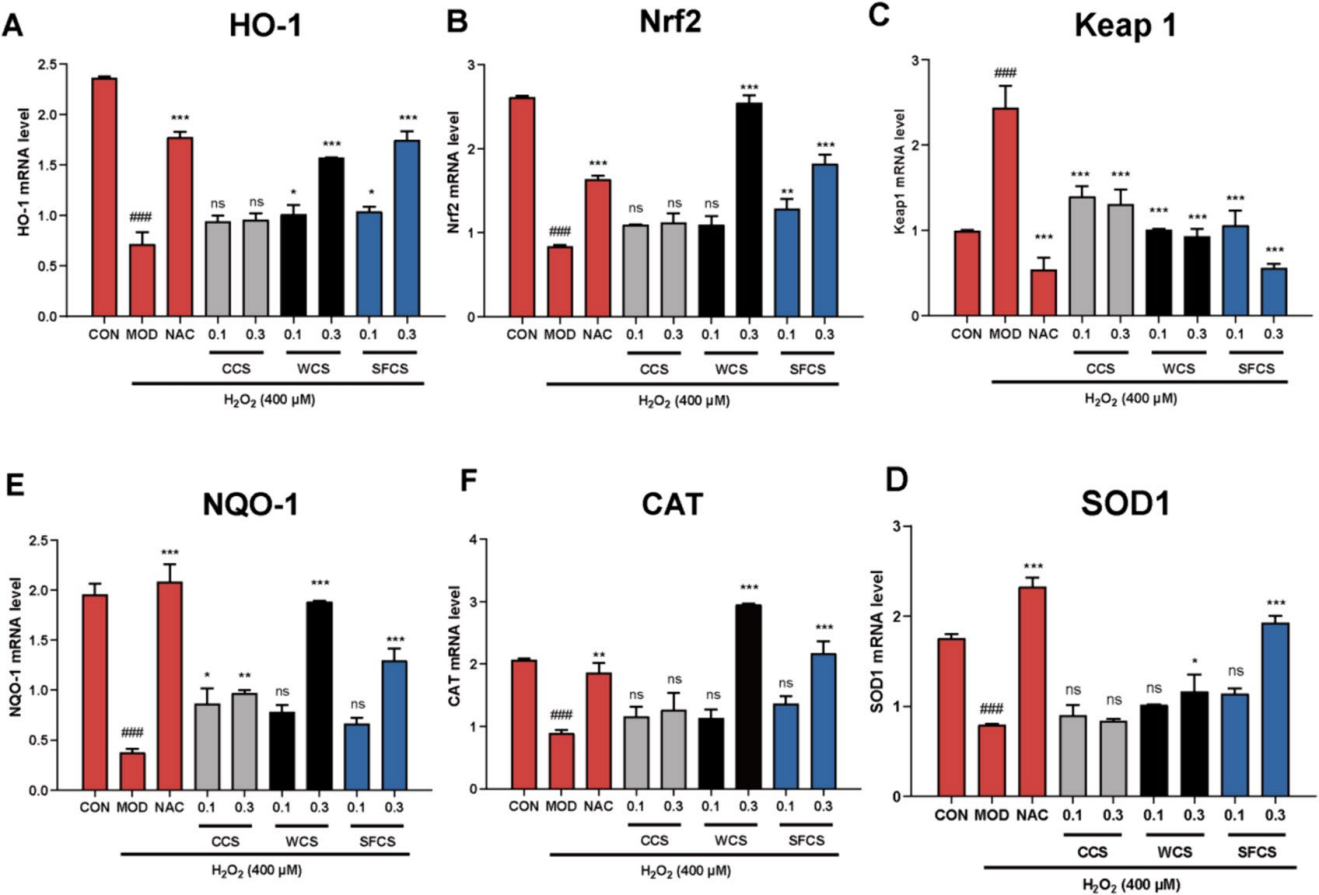
Effects of CCS, WCS, and SFCS on Nrf2-mediated antioxidant signaling transcripts were analyzed by qPCR. **A**–**F** HO-1, Nrf2, Keap 1, SOD1, CAT, and NQO-1 gene expression levels in response to CCS, WCS, and SFCS in the presence or absence of H_2_O_2_ treatment. Gene expression was normalized against β-actin. Data are means ± S.D. (*n* ≥ 3). ^###^*P* < 0.001 vs. CON group; ^*^P < 0.05, ^**^P < 0.01, ^***^P < 0.001 vs. MOD group; ns, not significantly different from the MOD group

**Fig. 8:**
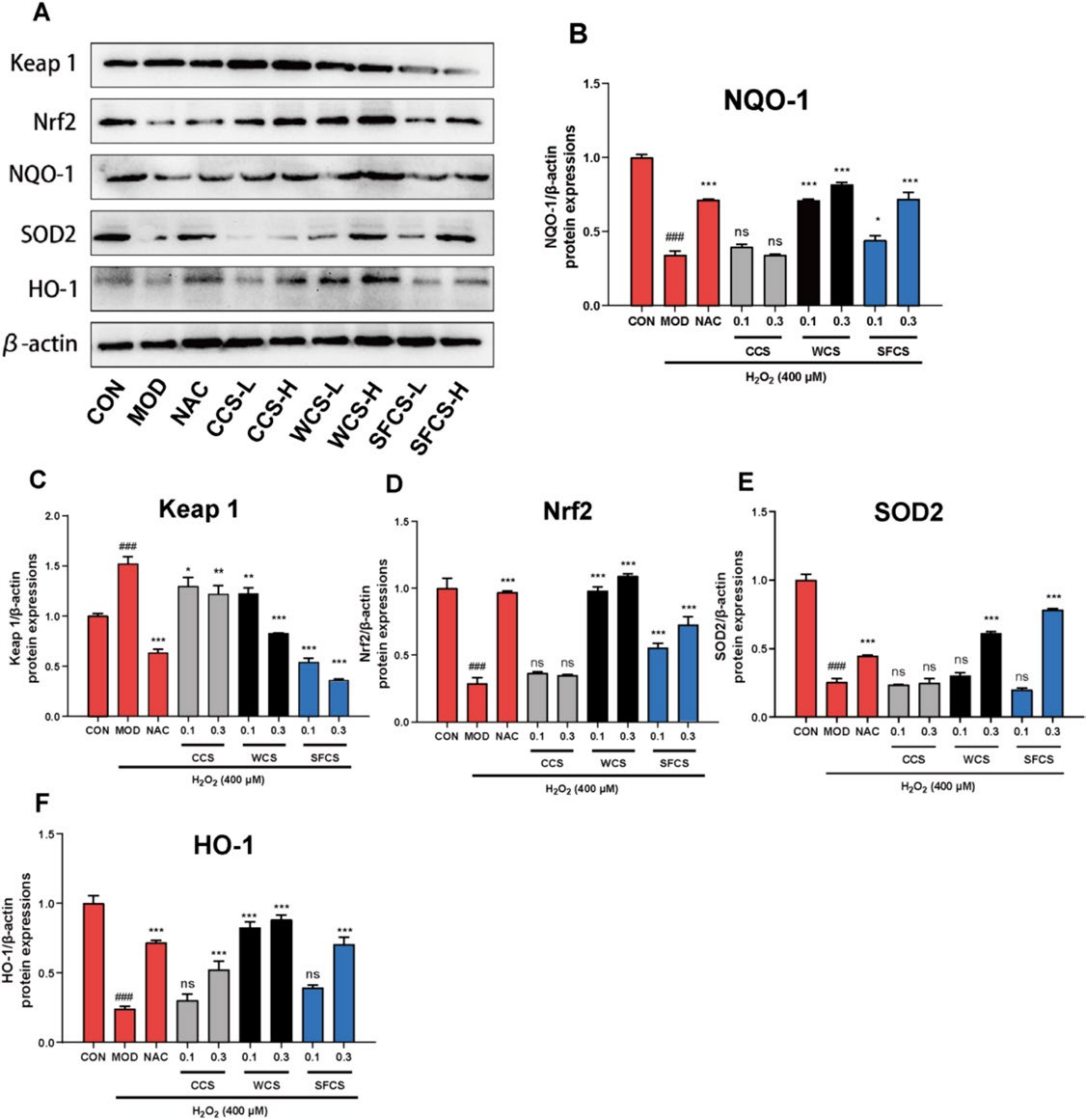
CCS, WCS, and SFCS regulate the Keap1/Nrf2 pathway in H_2_O_2_-challenged KGN cells. **A**–**F** NQO-1, Keap 1, Nrf2, SOD2, and HO-1 protein expression levels in response to CCS, WCS, and SFCS in the presence or absence of H_2_O_2_ treatment. Protein expression was normalized against β-actin. Data are means ± S.D. (*n* ≥ 3). ^###^*P* < 0.001 vs. CON group; ^*^P < 0.05, ^**^P < 0.01, ^***^P < 0.001 vs. MOD group; ns, not significantly different from the MOD group

The Keap1-Nrf2 antioxidant response element (ARE) signaling pathway regulates the transcription of various antioxidant genes [31]. Figure 8A–F show that H_2_O_2_ challenge upregulated Keap 1 protein and downregulated Nrf2, HO-1, SOD2, and NQO1 proteins. CCS, WCS and SFCS pretreatment had the opposite effects. The qPCR verified showed that CCS, WCS and SFCS pretreatment downregulated Keap1 mRNA and upregulated Nrf2, HO-1, CAT, SOD1, and NQO1 mRNA (Fig. 7A–F). The foregoing results suggest that CCS, WCS and SFCS inhibit the oxidative stress response in H_2_O_2_-challenged KGN cells by activating the Keap1-Nrf2/HO-1 pathway. Furthermore, WCS and SFCS more effectively counteracted the effects of oxidative stress than CCS. Thus, wine processing and stir-frying enhance the antioxidant efficacy of CS.

### Effects of CCS, WCS and SFCS on the PI3K-Akt pathway in H_2_O_2_-challenged KGN cells

**Fig. 9:**
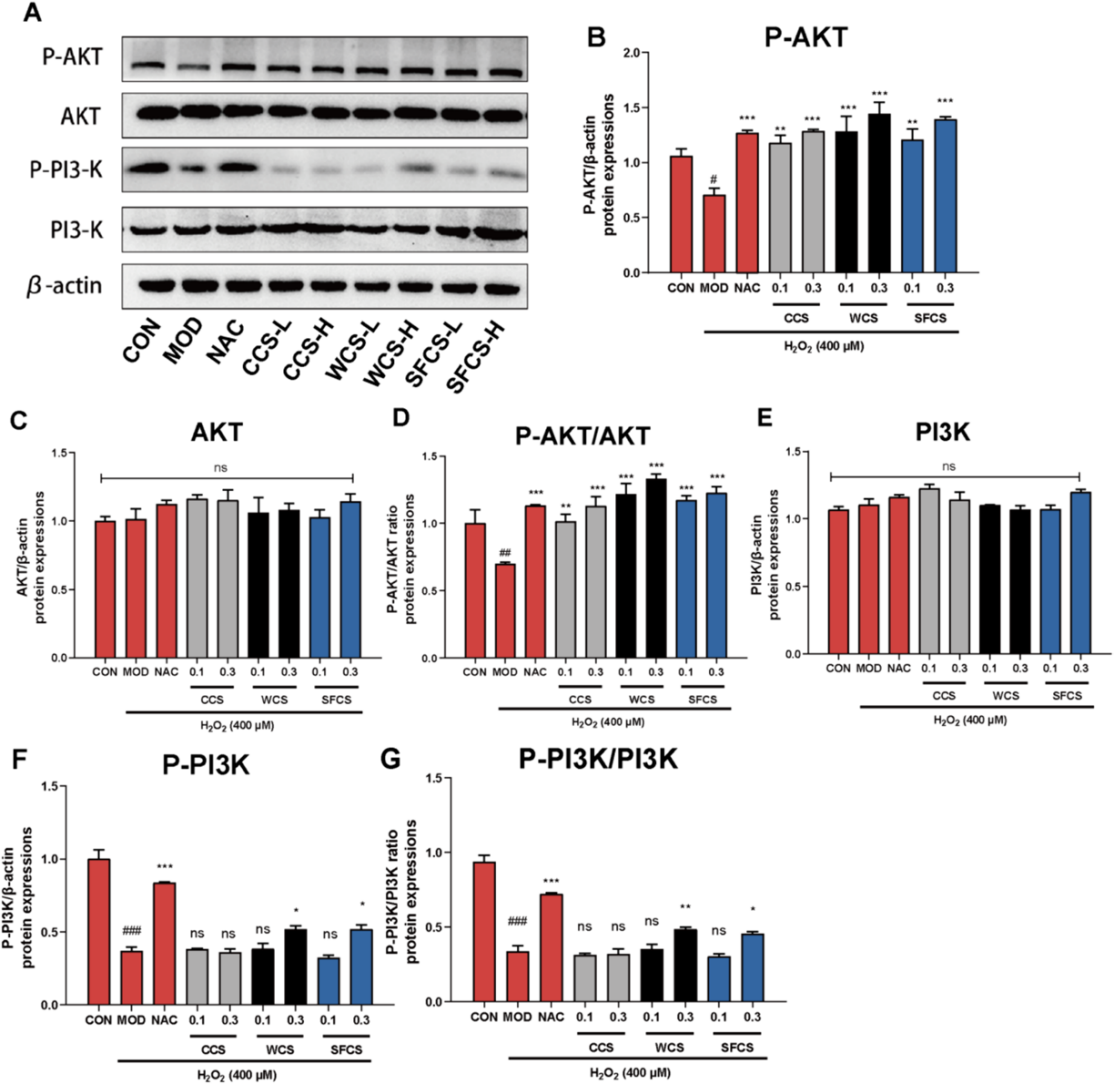
CCS, WCS, and SFCS regulate the Akt/PI3K pathway in H_2_O_2_-challenged KGN cells. **A**–**G** Akt, P-Akt, PI3K, and P-PI3K protein expression levels and P-Akt/Akt and P-PI3K/PI3K ratios in KGN cells treated with CCS, WCS, and SFCS in the presence or absence of H_2_O_2_ treatment. Protein expression was normalized against β-actin. Data are means ± S.D. (*n* ≥ 3). ^#^*P* < 0.05, ^##^*P* < 0.01, ^###^*P* < 0.001 vs. CON group; ^*^P < 0.05, ^**^P < 0.01, ^***^P < 0.001 vs. MOD group; ns, not significantly different from the MOD group

Numerous cellular stimuli and toxic insults activate the PI3K-Akt signaling pathway. PI3K catalyzes phosphatidylinositol-3,4,5-triphosphate (PIP3) biosynthesis in the cell membrane. In turn, PIP3 is a secondary messenger that activates Akt. The latter controls apoptosis, protein synthesis, metabolism, and the cell cycle by phosphorylating certain substrates associated with these processes [32]. Figure 9A–G show that the P-PI3K and P-Akt proteins were downregulated in H_2_O_2_-challenged KGN cells. Pretreatments with 0.1 mg/mL and 0.3 mg/mL CCS, WCS and SFCS upregulated P-PI3K and P-Akt in a dose-dependent manner. Nevertheless, there was no significant difference in total Akt or PI3K protein content. Hence, CCS, WCS and SFCS promote PI3K and Akt phosphorylation and activate the PI3K-Akt signaling pathway in KGN cells. However, 0.3 mg/mL WCS and SFCS were relatively more effective at inducing the PI3K-Akt signaling pathway in H_2_O_2_-challenged KGN cells than either CCS or the lower concentrations of WCS and SFCS.

## Discussion

Comprehensive research conducted over the past two decades has demonstrated that oxidative stress contributes to the initiation and development of female reproductive diseases such as premature ovarian insufficiency (POI). The latter is the most common fertility disorder in women below reproductive age [33]. The association between POI and redox imbalance has been reported in an earlier study [34]. The KGN cell line is a widely used in vitro POI model as it physiologically resembles the immature granulosa cells obtained from smaller antral follicles [35]. Modern pharmacology has indicated that Cuscutae Semen (CS) reverses reductions in testosterone levels and androgen receptor gene expression, and is immunomodulatory, hepatoprotective, antioxidant, anti-inflammatory, and anti-aging. It also enhances memory by inducing PC12 cell differentiation [8, 36, 37]. Crude Cuscutae Semen (CCS), wine-processed CS (WCS), and stir-frying-processed CS (SFCS) have different clinical applications as processing has conferred a unique pharmaceutical effect upon each of them according to traditional Chinese medicine (TCM). The results of component analysis showed that flavonoids, phenolic acids and lignans were the main differences between CCS, WCS and SFCS. The results were summarized in Fig. 1 and Table 2, which showed that the content and species of flavonoids changed greatly after wine-processed and stir-frying-processed.

However, few studies have been conducted on the therapeutic efficacy of CCS, WCS and SFCS against POI. To the best of our knowledge, the present study is one of the first to show that CCS, WCS and SFCS ameliorate apoptosis and oxidative stress in H_2_O_2_-challenged KGN cells by activating the Nrf2-Keap 1 signaling pathway and PI3K-Akt phosphorylation. Here, all concentrations of CCS, WCS and SFCS somewhat protected the KGN cells against H_2_O_2_-induced oxidative injury. Nevertheless, the antioxidant activity levels of WCS and SFCS were higher than that of CCS (Fig. 2). However, it remains to be determined whether WCS and SFCS have stronger antioxidant efficacy than CCS in H_2_O_2_-challenged KGN cells.

H_2_O_2_ exposure overloads cellular reactive oxygen species (ROS), depletes cellular reduced glutathione (GSH) pools, induces malondialdehyde (MDA) biosynthesis, and ultimately causes cell death [38]. Glutathione reductase (GR; EC 1.6.4.2) is a flavoprotein oxidoreductase that catalyzes the reduction of oxidized glutathione (GSSG) to GSH [39]. Here, we found that CCS, WCS and SFCS pretreatment decreased intracellular ROS levels in a dose-dependent manner (Fig. 4C and D). We also discovered that H_2_O_2_ exposure disrupted redox homeostasis because it increased MDA and GSSG and decreased SOD, T-AOC, GSH, GR, and GSH/GSSG. As NAC, CCS, WCS and SFCS partially reversed these abnormal changes, they might augment the ability of antioxidants to eliminate free radicals (Fig. 3). Taken together, the results of the present work suggest that although CCS, WCS and SFCS effectively alleviated oxidative stress in H_2_O_2_-challenged KGN cells, WCS and SFCS had superior efficacy to CCS.

ROS accumulation may cause oxidative stress leading to granulosa cell apoptosis, oocyte deterioration, and, by extension, reduced fertility [40]. Figure 2A shows that exposure to 400 μM H_2_O_2_ significantly increased the number of apoptotic cells. CCS, WCS and SFCS pretreatment significantly decreased apoptosis in a dose-dependent manner compared to the MOD group. The mitochondria-mediated intrinsic apoptotic pathway is triggered by a variety of endogenous or exogenous stimuli, including DNA damage, oxidative or endoplasmic reticulum stress, ischemia, and more. This pathway begins with the disruption of the mitochondrial membrane potential (MMP), which is followed by the release of cytochrome C and other proapoptotic proteins, the formation of an apoptosome, and ultimately, apoptosis. [41] The MMP collapses early in apoptosis [42]. This phenomenon was observed in the H_2_O_2_-challenged KGN cells. CCS, WCS and SFCS modulated apoptosis-related gene and protein expression (Figs. 5 and 6). Bcl-2 and Bax regulate apoptosis [43]. The Bax/Bcl-2 ratio determines the rate of this process [44] and cleaves and activates the apoptosis mediator cleaved caspase-9. The latter then cleaves and activates the apoptosis executor cleaved caspase-3 [45]. Here, CCS, WCS and SFCS significantly inhibited Bax, promoted Bcl-2, lowered the Bax/Bcl-2 ratio, and decreased the expression levels of cleaved caspase-9 and cleaved caspase-3 proteins. Figure 4A and B show that H_2_O_2_ treatment reduces MMP and aggravates apoptosis in KGN cells whereas CCS, WCS and SFCS partially reverse these changes. Hence, CCS, WCS and SFCS inhibit MMP loss and apoptosis in H_2_O_2_-challenged KGN cells via a mechanism involving Bcl-2, Bax, cleaved caspase-9, and cleaved caspase-3. As WCS and SFCS exerted these effects more strongly than CCS, however, the former two have greater anti-apoptotic efficacy than the latter in H_2_O_2_-challenged KGN cells.

The transcription factor (TF) Nrf2 regulates cellular oxidative stress [46, 47]. Under steady-state, it is localized mainly to the cytoplasm at a low basal protein level as it undergoes Keap1-mediated proteasomal degradation. During oxidative stress, electrophiles and ROS inactivate Keap 1 and liberate Nrf2 from the Nrf2-Keap1 complex. Thence, Nrf2 is translocated to the nucleus where it binds the antioxidant response element (ARE) and induces downstream antioxidant proteins such as HO-1 and NQO1 [46]. SOD1 is localized mainly to the cytosol whereas the mitochondrial antioxidant enzyme SOD2 detoxifies superoxide anion generated during oxidative phosphorylation [48]. Nrf2 activation ameliorates mitochondrial dysfunction and alleviates oxidative stress and aberrant hormone secretion in the ovaries [49]. The current study aimed to establish whether the Keap1-Nrf2/HO-1 pathway is implicated in the protective effects of CCS, WCS and SFCS on KGN cells. The western blot revealed that H_2_O_2_ exposure weakly upregulated Nrf2 in the KGN cells whereas CCS, WCS and SFCS downregulated Keap1 protein, strongly upregulated Nrf2, and promoted the nuclear translocation of the latter. CCS, WCS and SFCS also upregulated the antioxidant proteins HO-1, NQO1, and SOD2 (Fig. 8). The qPCR disclosed that CCS, WCS and SFCS also downregulated Keap1 mRNA but upregulated Nrf2, HO-1, CAT, SOD1, and NQO1 mRNA (Fig. 7). Altogether, these results demonstrated that CCS, WCS and SFCS alleviates apoptosis and oxidative stress via upregulation of Keap1-Nrf2/HO-1 signaling pathway-mediated antioxidant proteins, WCS and SFCS have a stronger effect on this pathway than CCS.

The PI3K/Akt signaling pathway regulates cell growth, proliferation, migration, invasion, apoptosis, and differentiation [50]. However, the pathway participating in Nrf2 induction is unknown. Earlier studies showed that the PI3K/Akt signaling pathways are involved in the upstream Nrf2 signal [51, 52]. Here, PI3K and Akt protein expression remain relatively unchanged. CCS, WCS and SFCS activated the PI3K/Akt pathway by upregulating P-Akt and P-PI3K protein expression (Fig. 9). Thus, these results indicated PI3K/Akt signaling pathway activation as the upstream signal stimulating Keap1-Nrf2/HO-1 in CCS, WCS and SFCS-treated POI cell model. Therefore, CCS, WCS and SFCS may have protected KGN cells against H_2_O_2_-induced oxidative stress by activating the PI3K-Akt signaling pathway in a dose-dependent manner, and WCS and SFCS were more effective than CCS in this regard.

## Conclusion

**Fig. 10:**
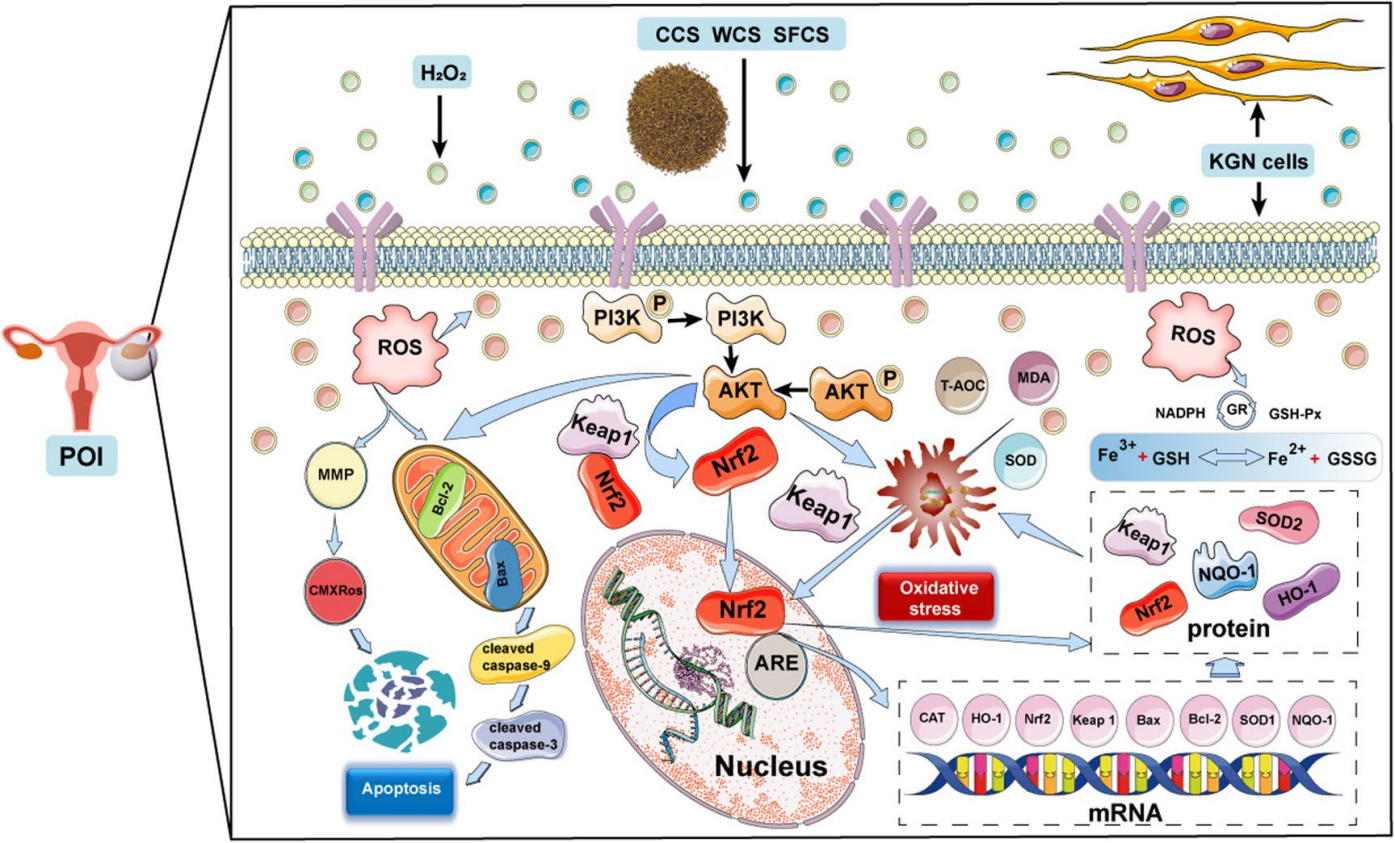
Mechanisms diagram of protective effects of CCS, WCS, SFCS in H_2_O_2_-induced KGN cells. CCS, WCS and SFCS inhibited apoptosis and oxidative stress in KGN cells subjected to H_2_O_2_ by activating the PI3K/Akt-mediated Keap1-Nrf2/HO-1 signaling pathway

**Fig. 11:**
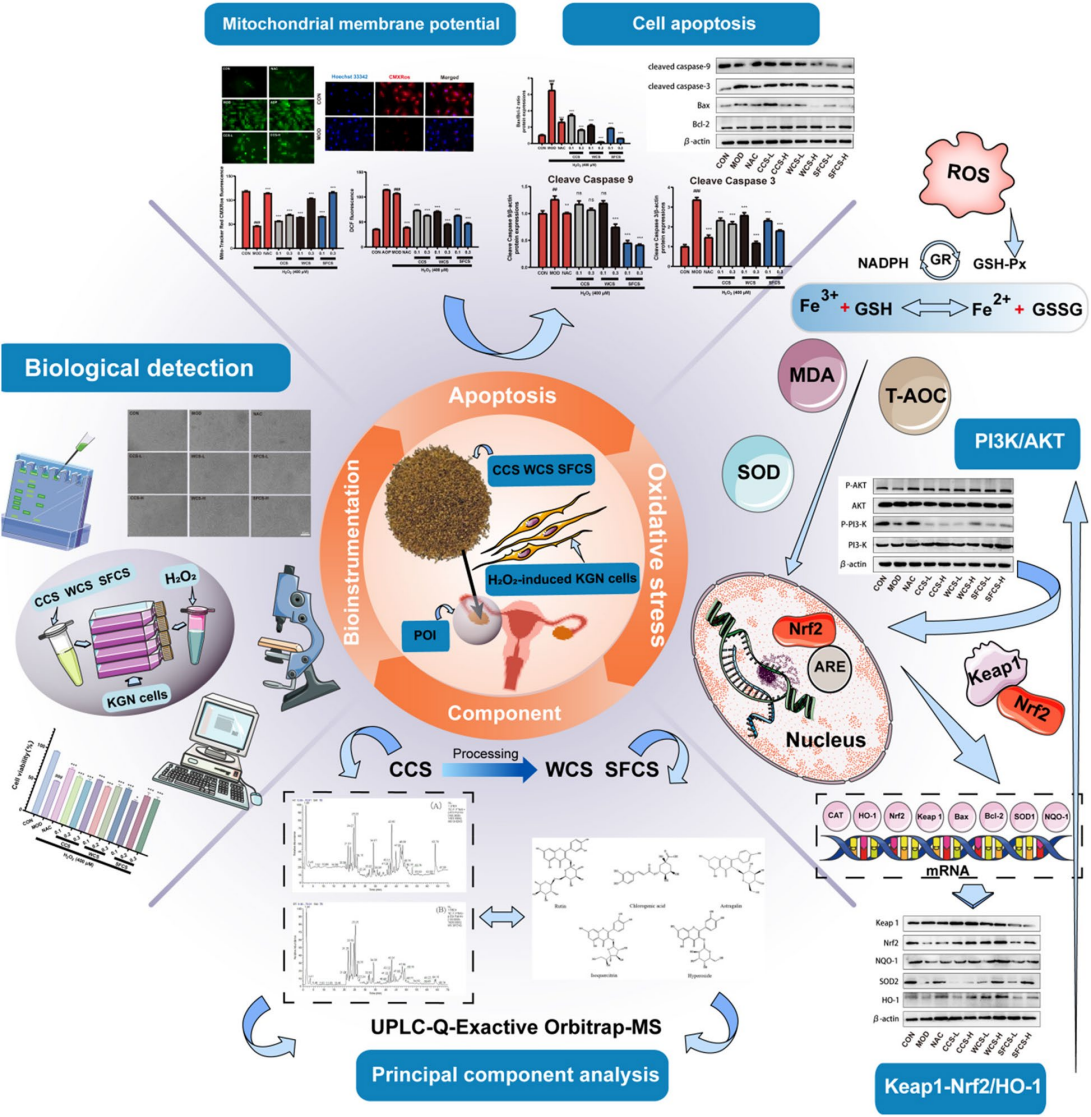
Graphical abstract

To the best of our knowledge, as showd in Figs. 10 and 11, the present study was one of the first to indicate that CCS, WCS and SFCS inhibited apoptosis and oxidative stress in KGN cells subjected to H_2_O_2_ by activating the PI3K/Akt-mediated Keap1-Nrf2/HO-1 signaling pathway. Moreover, this work showed that WCS and SFCS had superior efficacy to CCS in POI therapy and suggest that the relative efficacies of these formulations against this disorder should be evaluated using the appropriate in vivo models. Based on the study of POI, it is also possible to conduct ex vivo and in vivo binding studies of the common active monomer components of CCS, WCS and SFCS.

### Supplementary Information

Supplementary Material 1.

Supplementary Material 2.

## Data Availability

The datasets during and/or analysed during the current study available from the corresponding author on reasonable request.
