# Supplementary material for: Wine- and stir-frying processing of Cuscutae Semen enhance its ability to alleviate oxidative stress and apoptosis via the Keap 1-Nrf2/HO-1 and PI3K/AKT pathways in H2O2-challenged KGN human granulosa cell line
Source: BMC Complement Med Ther. 2024 May 15;24:189. doi: 10.1186/s12906-024-04491-5 (PMC11094956; doi:10.1186/s12906-024-04491-5)
Supplement: Supplementary file 2 — Supplementary Material 2. [file 12906_2024_4491_MOESM2_ESM.pdf]

Fig. 5 A

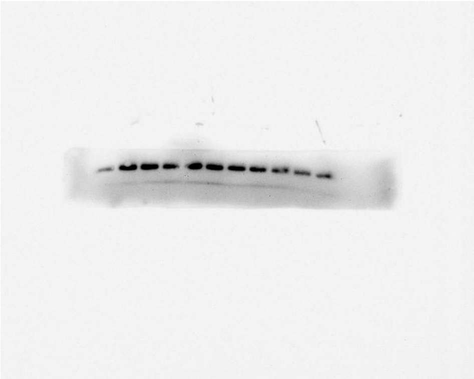

cleaved caspase-9

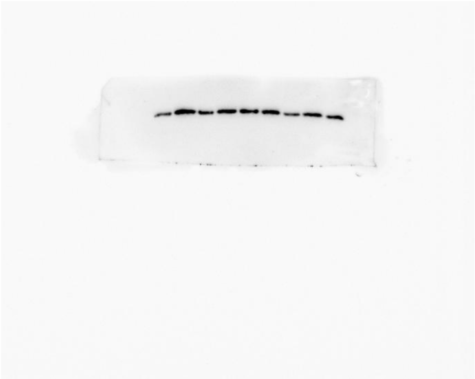

cleaved caspase-3

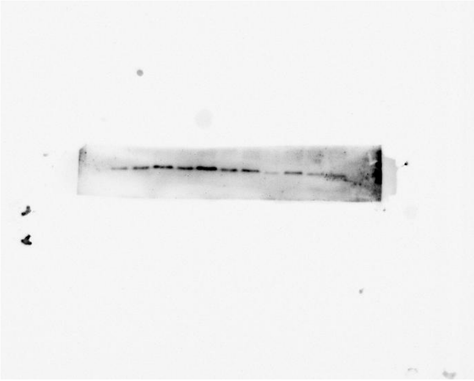

Bax

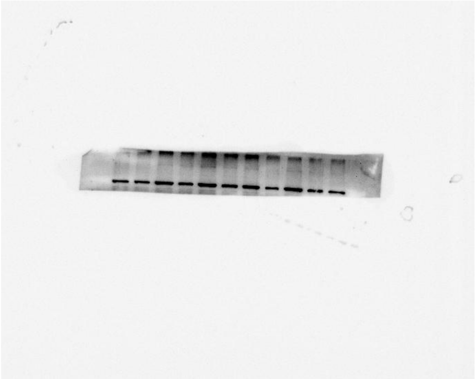

Bcl-2

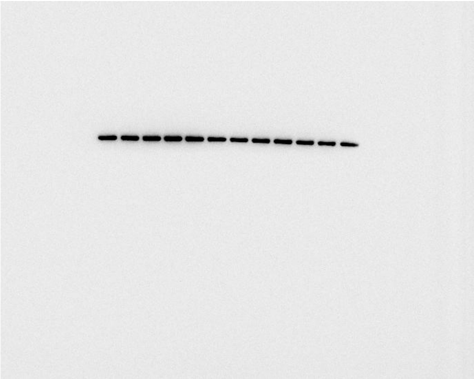

β -actin

Fig. 8 A

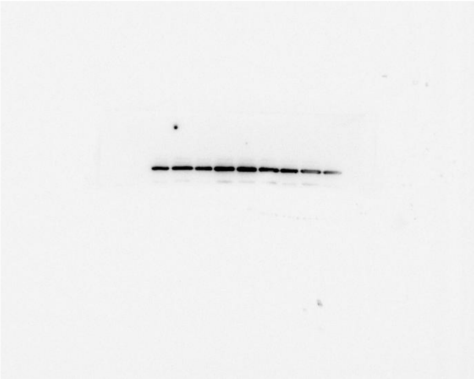

Keap 1

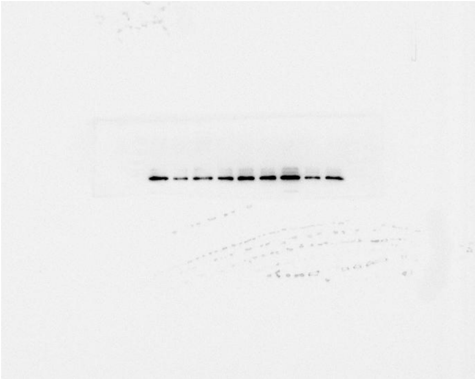

Nrf2

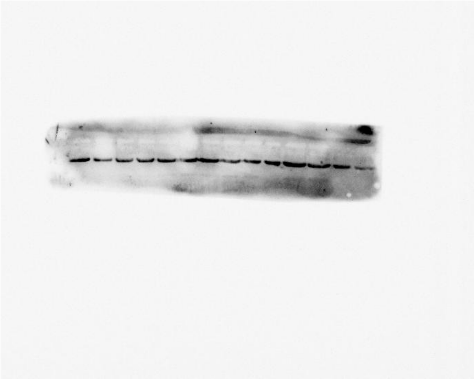

NQO-1

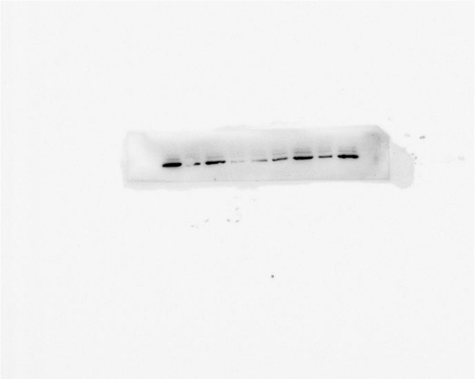

SOD2

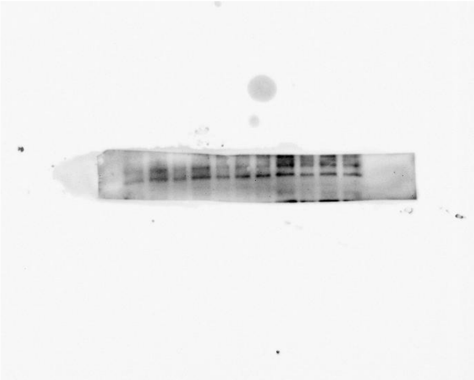

HO-1

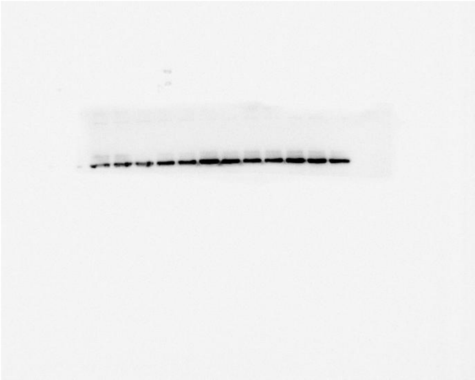

β -actin

Fig. 9 A

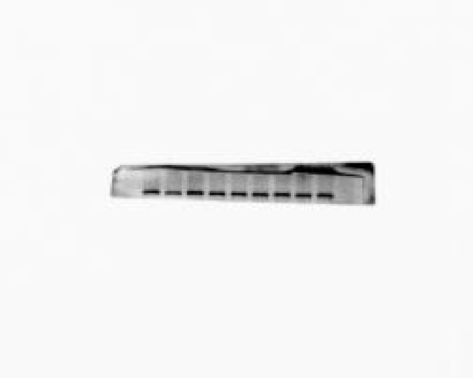

P-AKT

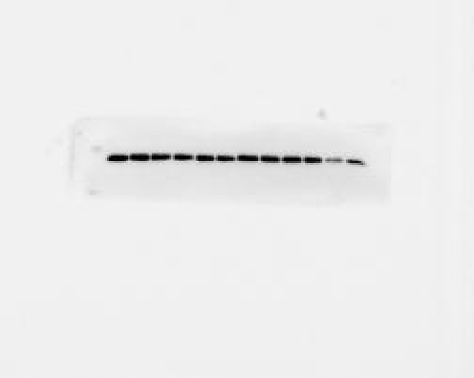

AKT

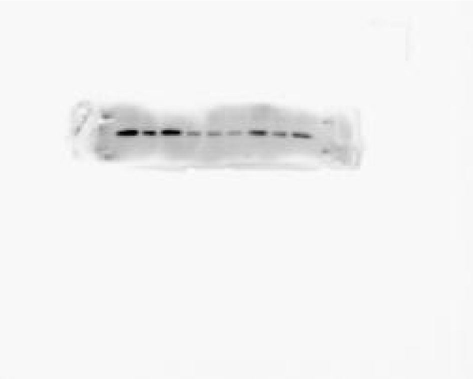

P-PI3-K

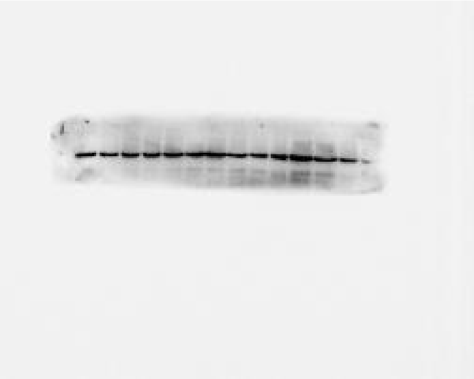

PI3-K

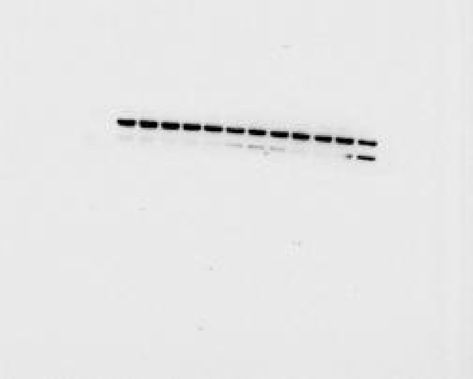

β -actin
